# Molecular Data Confirm Interspecific Limits of Four *Alloxysta* and One *Phaenoglyphis* Species of Parasitic Wasps within the Subfamily Charipinae (Cynipoidea: Figitidae)

**DOI:** 10.3390/insects15050354

**Published:** 2024-05-14

**Authors:** Mar Ferrer-Suay, Mariana Bulgarella, George E. Heimpel, Ehsan Rakhshani, Jesús Selfa

**Affiliations:** 1 Departament de Zoologia, Facultat de Ciències Biològiques, Universitat de València, Campus de Burjassot-Paterna, Dr. Moliner 50, E-46100 Burjassot (València), Spain; jesus.selfa@uv.es; 2 School of Biological Sciences, Victoria University of Wellington, Wellington 6012, New Zealand; mariana.bulgarella@vuw.ac.nz; 3 Department of Entomology, University of Minnesota, St. Paul, MN 55108, USA; heimp001@umn.edu; 4 Department of Plant Protection, College of Agriculture, University of Zabol, Zabol P.O. Box 98615-538, Iran; rakhshani@uoz.ac.ir

**Keywords:** *Alloxysta*, Charipinae, hyperparasitism, molecular markers, *Phaenoglyphis*, species boundaries

## Abstract

**Simple Summary:**

The interspecific limits within the Charipinae subfamily are still not clear, although many improvements have been made to the taxonomy of this group. Three genes (mitochondrial COI and 16S rDNA, and nuclear ITS2 rDNA) have been sequenced from five different species: *Alloxysta brevis* (Thomson, 1862), *A. castanea* (Hartig, 1841), *A. ramulifera* (Thomson, 1862), *A. victrix* (Westwood, 1833), and *Phaenoglyphis villosa* (Hartig, 1841). The phylogeny resulting from concatenating the three genes confirms that these molecular markers can be used to separate Charipinae species and also shows the close relationship between the genera *Alloxysta* and *Phaenoglyphis*. The genetic distances of these species are presented.

**Abstract:**

The hymenopteran subfamily Charipinae (Cynipoidea: Figitidae) consist of a group of parasitic wasps that are exclusive hyperparasitoids of Hemipteran. The species boundaries in Charipinae have historically been unclear. While diagnostic morphological features have been established for the stepwise separation of species, it is recommended to confirm those limits using molecular data. Here, we focus on the genera *Alloxysta* Förster, 1869 and *Phaenoglyphis* Förster, 1869, both of which contain species that are hyperparasitoids of aphids. We sequenced three genes (mitochondrial COI and 16S rDNA, and nuclear ITS2 rDNA) from specimens that were identified as belonging to five species: *Alloxysta brevis* (Thomson, 1862), *A. castanea* (Hartig, 1841), *A. ramulifera* (Thomson, 1862), *A. victrix* (Westwood, 1833), and *Phaenoglyphis villosa* (Hartig, 1841). The phylogeny resulting from concatenating these genes supported the species status of the five morphologically identified taxa, with *P. villosa* nested within *Alloxysta*. Our study thus indicates that these molecular markers can successfully distinguish charipine species, and also indicates that the genera *Alloxysta* and *Phaenoglyphis* may be more closely related than previously hypothesized. We also present the first estimates of genetic distances for these species. Future studies that include more species, loci, and/or genomic data will complement our research and help determine species relationships within the Charipinae subfamily.

## 1. Introduction

Hymenopteran parasitoids are important in the biological control of pests [1,2,3,4,5,6]. However, biological control scientists often experience difficulty identifying specimens to the species level, and some biological control projects are further complicated by the presence of cryptic species [7,8,9]. There is therefore a great opportunity to improve the understanding of biological control interactions with the increased ability to incorporate molecular tools for identification of parasitoid species [10]. Members of the hymenopteran subfamily Charipinae (Cynipoidea: Figitidae) are secondary parasitoids (hyperparasitoids) of aphids via Aphidiinae (Braconidae) and Aphelinidae (Chalcidoidea), and of psyllids via Encyrtidae (Chalcidoidea) [11]. Charipine hyperparasitoids frequently attack biological control agents [12,13,14,15,16], and the suppression exerted on the populations of primary parasitoids has been demonstrated in greenhouses [17,18] and likely occurs in the field as well [11]. Thus, taxonomic studies within the Charipinae are important from the standpoint of biological control programs, in which accurate species identification can be problematic.

The effectiveness of molecular methods of species identification has led to a proposal of standardization and large-scale application whose purpose is to inventory as much life on the planet as possible: “The Barcode of Life” [19], this is a technique for characterizing species of organisms using a short DNA sequence from a standard and agreed-upon position in the genome. The cytochrome c oxidase subunit 1 mitochondrial region (COI) has emerged as the standard barcode region for higher animals [19]. This sequence is practically the same size in all animals, and a fragment of 650 nucleotides typically encompasses enough variation to allow discernment between two closely related species. It has been proposed that taxonomists continue with the morphological and ecological work they have been doing, but that they also include a DNA barcode sequence to allow non-specialists to quickly recognize species [19,20,21,22,23,24]. A major advantage of the barcode approach is that sequences of the COI region obtained from unidentified specimens can be compared with existing DNA sequence databases for identification. Also, COI sequences could be used to identify new or cryptic species. In addition, some authors argue that the evolution of COI is sufficiently rapid to investigate intraspecific diversity in some taxa [21]. Another mitochondrial gene, 16S ribosomal RNA encoding gene sequences (16S rDNA), is extensively used for phylogenetic analysis, and it complements COI for species-level identification. The nuclear-encoded internal transcribed spacer 2 (ITS2 rDNA) has been used for species-level identification alone [25] or in combination with other markers [26,27,28].

There is a very limited number of studies on the molecular identification of *Alloxysta* and *Phaenoglyphis* species. To the best of our knowledge, the only molecular study outside our research group aimed to differentiate two species of hyperparasitoids, *Alloxysta xanthopsis* (Ashmead 1896) and *Dendrocerus carpenteri* (Curtis, 1829) [29]. The majority of previous studies have focused on establishing species limits of charipines using DNA barcoding [30,31]. *Alloxysta* (Figitidae, Charipinae) is the most abundant and widely distributed genus within the subfamily and found on every continent, except for Antarctica [32]. There are few diagnostic features that allow species delimitation within *Alloxysta*, and even those available can be very difficult to recognize for the non-expert. The identification to species level has always been very difficult for this genus [33]. *Alloxysta* species have been found attacking numerous host species [34], and host specificity can vary greatly among species [35]. *Phaenoglyphis* is the second most diverse genus within Charipinae. Specimens of *Phaenoglyphis* are rarely encountered in the field, so information about this genus is more limited. The first molecular characterization of seven *Phaenoglyphis* species using COI sequences was conducted recently. The delimitation of these seven species was largely congruent with their morphological identifications [36].

The aim of this study is to corroborate the morphological identification of five charipine species from previous studies [30,31] with molecular data. We amplified three genes (COI, ITS2, and 16S) for individuals that we identified morphologically as belonging to five species: *Alloxysta brevis* (Thomson 1862), *A. castanea* (Hartig 1841), *A. ramulifera* (Thomson 1862), *A. victrix* (Westwood 1833), and *Phaenoglyphis villosa* (Hartig 1841). 

## 2. Materials and Methods

### 2.1. Morphological Identification

The specimens of *Alloxysta* and *Phaenoglyphis* were collected from Yprès, Belgium (*n* = 35) during the 2022 summer season as part of another project. A total of 1025 specimens were identified using morphological characters and a published dichotomous key [37]. Thirty-five of these specimens preserved in absolute ethanol were selected from five species (seven of each): *Alloxysta brevis*, *A. castanea*, *A. ramulifera*, *A. victrix*, and *Phaenoglyphis villosa*. These 35 specimens were examined using a stereo microscope (OPTIKA ZSM-2, Ponteranica, Italy,) and environmental scanning electron microscope (FEI Quanta 200 ESEM) at the scientific technical services of the University of Barcelona and Hitachi S4800 from SCIE (Servicio Central de Soporte a la Investigación) at the Universitat de València. 

### 2.2. DNA Extraction, PCR Amplification, and Sequencing

DNA extraction, PCR, and Sanger sequencing were performed by AllGenetics & Biology SL (www.allgenetics.eu, accessed on 27 February 2024). DNA extraction was performed non-destructively using whole specimens with the Quick-DNA Microprep Plus Kit (Zymo Research, Irvine, CA, USA), following the manufacturer’s instructions. Briefly, the entire specimen was submerged in an extraction buffer without smashing or breaking it up. The kit employs a combination of enzymatic and chemical extraction mechanisms. This DNA extraction method leaves the insect integument intact so that the specimen can be morphologically studied. DNA was eluted in 12 μL of elution buffer. DNA was quantified using a Qubit High-Sensitivity dsDNA Assay (Thermo Fisher Scientific, Waltham, MA, USA). Sample yield was very low (see results). Three different genes were PCR-amplified: the mitochondrial cytochrome oxidase I gene (COI) and 16S ribosomal RNA encoding gene sequences (16S rDNA), and the nuclear internal transcribed spacer 2 (ITS2 rDNA). 

PCR protocols varied for each gene as follows. For COI, we used the forward primer LCO1490 paired with the reverse primer HCO2198 [38]. PCRs were carried out in 12.5 μL volumes, containing 2 μL of template DNA solution, 0.5 μM of each primer, 6.25 μL of Supreme NZYTaq 2× Green Master Mix (NZYtech, Lisboa, Portugal), and ultrapure water. The reaction mixture was incubated as follows: an initial denaturation step at 95 °C for 5 min, followed by 35 cycles of 95 °C for 30 s, 49 °C for 45 s, 72 °C for 45 s, and a final extension step at 72 °C for 7 min. Samples that showed weak products were reamplified successfully. These PCRs were carried out in a final volume of 25 μL, containing 10 μL of the first PCR product, 0.5 μM of the primers, 6.25 μL of Supreme NZYTaq 2× Green Master Mix, and ultrapure water up to 25 μL. The reaction mixture was incubated as follows: an initial denaturation step at 95 °C for 5 min, followed by 5 cycles of 95 °C for 30 s, 49 °C for 45 s, 72 °C for 45 s, and a final extension step at 72 °C for 7 min. 

For 16S, the forward primer 16SWbF [24] was paired with the reverse primer 16SWbR [39] to amplify a fragment around 500 bp long. The PCR reactions were carried out in a final volume of 12.5 μL, containing two slightly different reaction mixtures: (a) 6.25 μL of Supreme NZYTaq 2× Green Master Mix, 0.5 μM of the primers, 2 μL of template DNA solution, and ultrapure water up to 12.5 μL and (b) 3.13 μL of Supreme NZYTaq 2× Green Master Mix, 0.5 μM of the primers, 1.25 μL of template DNA, and ultrapure water up to 12.5 μL. Thermal cycling conditions included an initial denaturation step at 95 °C for 5 min, followed by 35 cycles of 95 °C for 30 s, 49 °C/51 °C (for different reaction mixtures: a and b, respectively) for 45 s, 72 °C for 45 s, and a final extension step at 72 °C for 7 min. 

For ITS2, the primer pair used was ITS2F and ITS2R [25] which amplifies a region 400 bp long. PCRs were carried out in a final volume of 12.5 μL, containing 2 μL of template DNA solution, 0.5 μM of the primers, 6.25 μL of Supreme NZYTaq 2× Green Master Mix, and ultrapure water up to 12.5 μL. Cycling conditions included an initial denaturation step at 95 °C for 5 min, followed by 35 cycles of 95 °C for 30 s, 57 °C for 45 s, 72 °C for 45 s, and a final extension step at 72 °C for 7 min.

A negative control that contained no DNA was included in every PCR round to check for cross-contamination. PCR products were run on 2% agarose gels stained with GreenSafe (NZYtech, Lisboa, Portugal) and imaged under UV light to verify the amplicon size. The PCR products were bi-directionally sequenced on an ABI 3730xl DNA Analyzer (Applied Biosystems, Waltham, MA, USA), with the same primers as those used in the PCR amplification. The amplification products that showed an intense background smear, primer dimers, or non-specific bands in the electrophoresis gel were purified using magnetic beads (Mag-Bind, Omega-Biotek, Norcross, GA, USA) prior to sequencing.

Initial electropherogram analysis was conducted by AllGenetics & Biology SL in Geneious 8.1.9 (https://www.geneious.com, accessed on 7 November 2023). The primer annealing regions and the low-quality regions at both ends of each electropherogram were trimmed (error probability limit of 0.03). Sequence reads were manually checked for sequencing errors or ambiguous base calls. Ambiguities and polymorphic positions were coded using the IUPAC ambiguity code.

### 2.3. Phylogenetic Analyses

The sequences were aligned for each gene region using the Geneious Prime v.2023.2.1 alignment algorithm with the default parameter settings (Biomatters, Auckland, New Zealand). The final length of COI retained for analyses was 612 bp, and all 35 individuals were amplified successfully. A region of 352 bp of 16S was amplified successfully for 24 specimens. For ITS2, a 415 bp region was retained for 28 individuals. We concatenated the three gene regions and included a COI sequence from a cynipid gall wasp, *Barbotinia oraniensis* (Barbotin) (GenBank accession number AF395179.1), as the outgroup to obtain a final alignment consisting of 1374 bp for 36 individuals (Supplemental Data File S1).

As previously found, *P. villosa* presented a 6 bp deletion in COI (Ferrer-Suay et al. 2018) at position 467–472 in our alignment, not present in any of the four *Alloxysta* species. For 16S and ITS2, the five species presented multiple gaps of variable lengths. We implemented the gap-coding program FastGap 1.2 (University of Aarhus, Aarhus, Denmark) [40] for the data on the three concatenated loci. This software codes gaps using the method of Simmons and Ochoterena [41], and gaps are added to the data file as separate partitions. In this case, the resulting alignment consisted of 1471 bp for the 36 individuals included (Supplemental Data File S2).

We built phylogenetic trees using the MrBayes v.3.2.6 plug-in [42] for Geneious Prime. Clade probabilities were obtained from the posterior distribution. Bayesian analyses were replicated twice, each with four Markov chains of 1.5 million generations, using a general time-reversible model and gamma-distributed rate variation, with a proportion of invariant sites (GTR + G + I). Trees were sampled every 1000 generations, and the first 500,000 generations were discarded as burn-in. We built phylogenetic trees for the original alignment with gaps uncoded (1374 bp) and for the alignment with gaps coded as separate partitions at the end (1471 bp). Sequences generated in this study have been archived in GenBank (accession numbers for COI: PP097396-PP097430, for 16S: PP109087-PP109110, and for ITS2: PP102679-PP102706).

Next, we downloaded all COI sequences available for *Alloxysta brevis*, *A. castanea*, *A. ramulifera*, *A. victrix*, and *Phaenoglyphis villosa* from GenBank. These sequences were aligned to the COI alignment we generated in this study and included *Barbotinia oraniensis* as the outgroup. The resulting alignment consisted of 615 bp long sequences for 239 individuals (Supplemental Data File S3). We used AIC [43] as implemented in Modeltest 3.7 [44] for PAUP* 4.0 [45] to determine the model of sequence evolution that best fits the mitochondrial COI data. The selected model was HKY85 + I + G. We obtained clade probabilities from the posterior distribution using MrBayes with four Markov chains of 1 million generations each. Trees were sampled every 1000 generations, and the first 500,000 generations were discarded as burn-in.

We calculated the intra- and interspecific pairwise distances between sequences for COI. Analyses were conducted using the Kimura 2-parameter model [46]. This analysis included 35 nucleotide sequences. All ambiguous positions were removed for each sequence pair (pairwise deletion option). The final dataset included 532 positions. Evolutionary analyses were conducted in MEGA11 [47]. We present the mean ± standard deviation of intra- and interspecific distances per species.

## 3. Results

**Figure 1:**
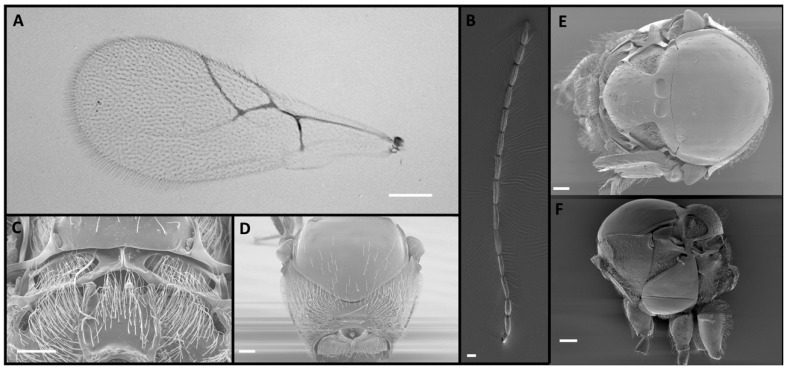
*Phaenoglyphis villosa* (Hartig, 1841), male: (**A**) forewing, with radial cell partially open; (**B**) antenna; (**C**) propodeum; (**D**) pronotum; (**E**) mesoscutum, with scutellar foveae marked with an arrow; (**F**) mesosoma, lateral view, with mesopleural sulcus marked with an arrow. Photographic credit: Jordi Paretas-Martínez. Scale: 50 μm.

**Figure 2:**
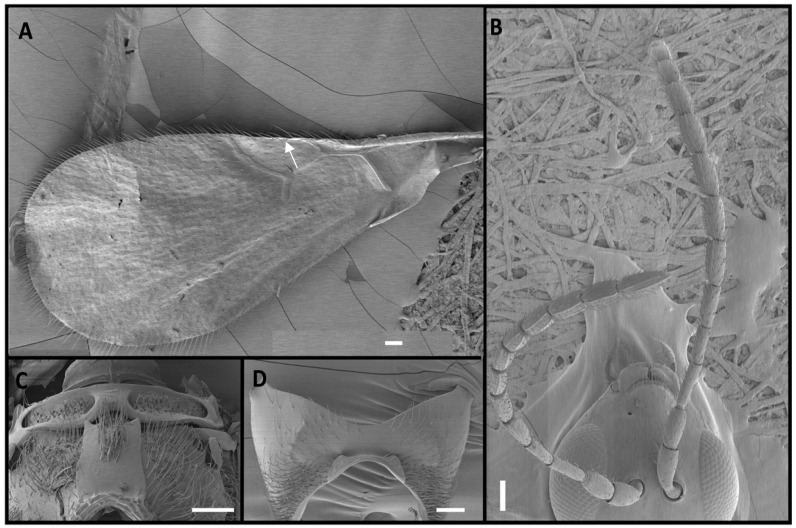
*Alloxysta castanea* (Hartig, 1841), female: (**A**) forewing, with radial cell partially open, marked with an arrow; (**B**) antenna; (**C**) propodeum; (**D**) pronotum. Photographic credit: Mar Ferrer-Suay. Scale: 50 μm.

**Figure 3:**
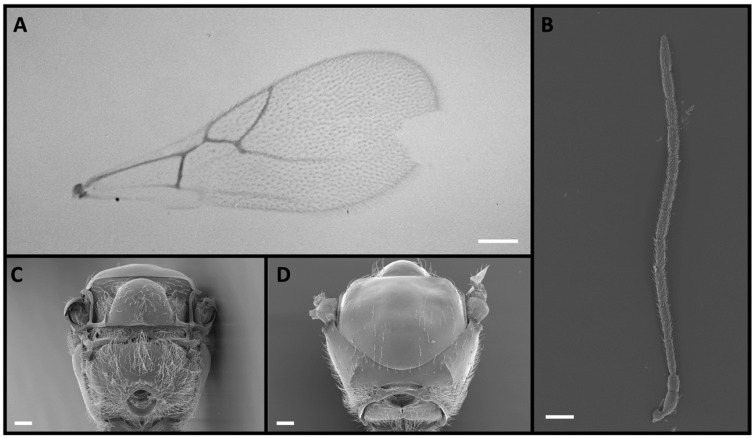
*Alloxysta victrix* (Westwood, 1833), female: (**A**) forewing, with closed radial cell; (**B**) antenna; (**C**) propodeum; (**D**) pronotum. Photographic credit: Jordi Paretas-Martínez. Scale: 50 μm.

**Figure 4:**
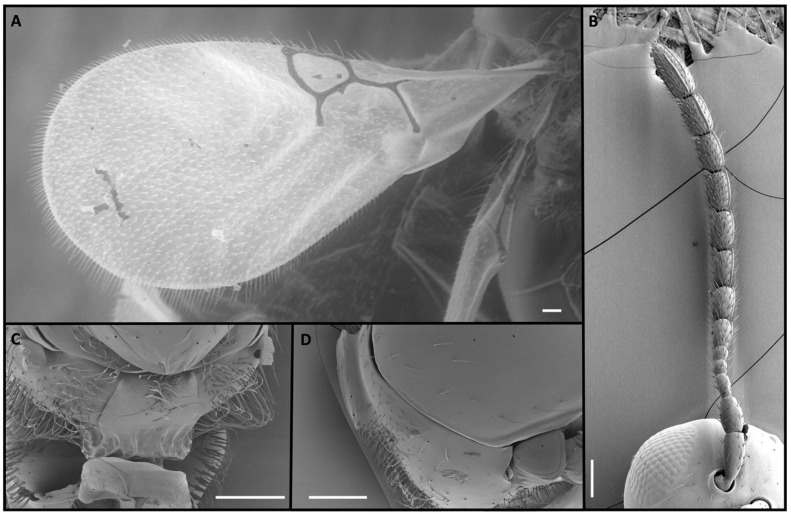
*Alloxysta brevis* (Thomson, 1862), female: (**A**) forewing; (**B**) antenna; (**C**) propodeum; (**D**) pronotum. Photographic credit: Mar Ferrer-Suay. Scale: 50 μm.

**Figure 5:**
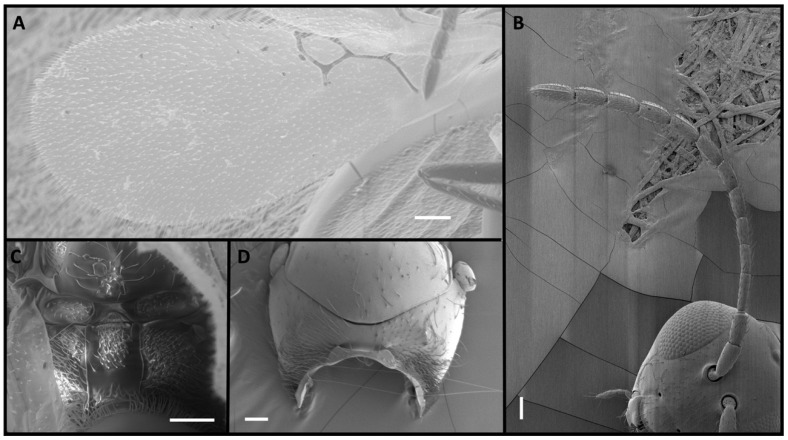
*Alloxysta ramulifera* (Thomson, 1862), female: (**A**) radial cell; (**B**) antenna; (**C**) propodeum; (**D**) pronotum. Photographic credit: Mar Ferrer-Suay. Scale: 50 μm.

Four *Alloxysta* and one *Phaenoglyphis* species were compared in this study. These five species can be distinguished by an expert based on five morphological characters. The genus *Phaenoglyphis* is currently being revised, and it is well differentiated from *Alloxysta* by the mesopleural sulcus and the scutellar foveae present (Figure 1). *Phaenoglyphis villosa* can be differentiated from other species of *Phaenoglyphis* by its partially open radial cell and mesoscutum without notauli. *Alloxysta castanea* has radial cells partially open (Figure 2), *A. victrix* does not have propodeal carinae (Figure 3), and *A. brevis* does not have pronotal carinae (Figure 4). *Alloxysta ramulifera* has a combination of features not present in the other *Alloxysta* species (Figure 5).

The following is a key to differentiate between these five species:

1. Lower part of mesopleuron with horizontal sulcus (Figure 1F). ……………. *P. villosa*

– Mesopleuron lacks horizontal sulcus. …………………………………………...……..... 2

2. Radial cell partially open (Figure 2A). ……………………………………….. *A. castanea*

– Radial cell closed. …………………………………………………………………………... 3

3. Propodeal carinae absent (Figure 3C). …………………………………………. *A. victrix*

– Propodeal carinae present, forming a plate (Figure 4C and Figure 5C)..…………….. 4

4. Pronotal carinae absent; F1 shorter than pedicel (Figure 4B). ……………….. *A. brevis*

– Pronotal carinae present; F1 longer than pedicel (Figure 5B). …………… *A. ramulifera*

In this study, we sequenced three partial molecular markers. For COI, all 35 specimens were amplified satisfactorily, although some samples needed to be reamplified to obtain the sequence of interest. For ITS2, 28 out of the 35 specimens produced good sequences. For 16S, we only obtained 24 sequences and no individual of *A. ramulifera* amplified via PCR, suggesting that species-specific primers need to be re-designed to amplify this locus in this species. The COI sequences presented 89.6% pairwise identity, and 74.5% of the 612 bp sites were identical. ITS2 presented 71.2% pairwise identity, and 37.4% of the sites were identical out of 415 bp. The 16S sequences presented 87.4% pairwise identity, with 70.2% of the sites being identical out of 352 bp. PCR amplification of these loci was not straightforward for these specimens. For example, a nested PCR approach was required to obtain 612 bp of COI of good quality. The primers for 16S amplify a fragment around 500 bp long, but we had to trim sequences to 352 bp to retain only good-quality base calls. We believe these difficulties are likely due to the extremely low concentrations of the DNA extractions (no specimen rendered > 3 ng/μL of DNA). These wasps are extremely small (1–2 mm), providing very limited starting material. Using the whole specimen material mechanically destroyed by a homogenizer is necessary to extract enough quantity of DNA for the subsequent studies.

**Figure 6:**
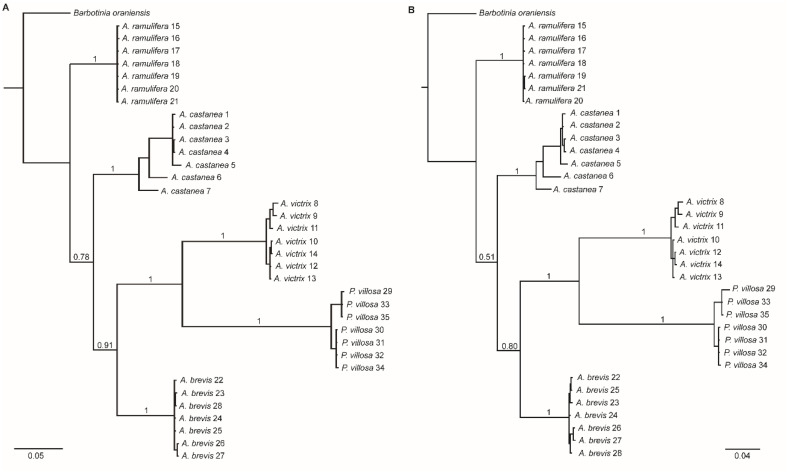
Bayesian phylogenies for four *Alloxysta* species and *Phaenoglyphis villosa*, based on concatenated analysis of three gene regions (**A**) with gaps not coded as separate characters. (**B**) with gaps coded as separate characters. Trees are rooted with *Barbotinia oraniensis* as the outgroup. Support for clades is indicated by the posterior probability values above branches.

The phylogenetic tree resulting from the three concatenated genes (when gaps were not coded as separate characters, 1374 bp alignment) shows that the 35 specimens studied separate into five monophyletic groups corresponding to the five species to which they were assigned based on morphology, and that the molecular markers combined can successfully distinguish charapine species. Considering these five species, *Alloxysta victrix* is the sister taxon to *P. villosa*, and this clade is more closely related to *A. brevis*, with *A. castanea* being more closely related to these three species than *A. ramulifera* (Figure 6A). It is worth highlighting the fact that *P. villosa* being nested within *Alloxysta* is an unexpected result. *Phaenoglyphis villosa* belongs in a different genus, based on morphological differences [32]. The Bayesian tree resulting from the concatenated sequences 1471 bp long with gaps coded as separate partitions uncovered the same relationships between the five species as the tree built with gaps not coded, albeit with lower posterior probability support (Figure 6B).

**Figure 7:**
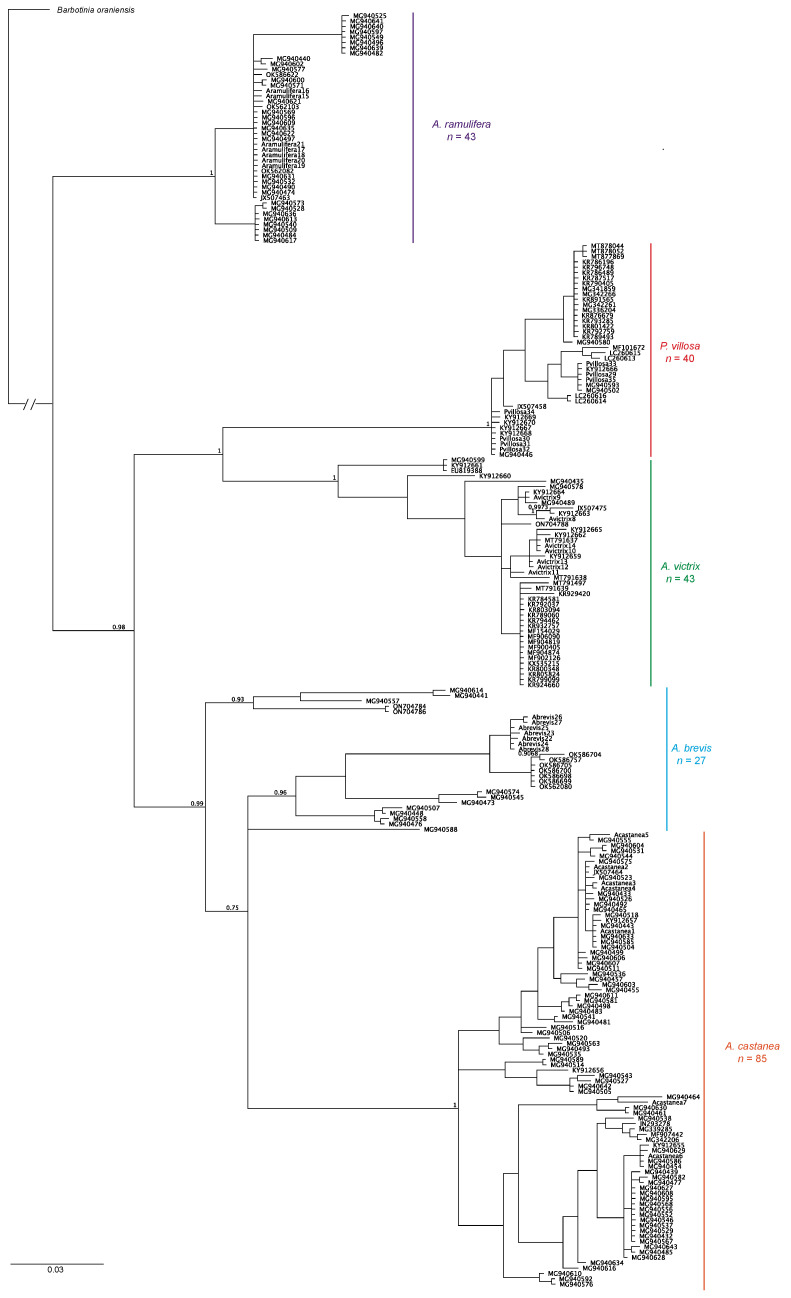
Bayesian phylogeny constructed combining COI sequences from the 35 individual wasps from this study (individuals labeled *Abrevis*, *Acastanea*, *Aramulifera*, *Avictrix*, and *Pvillosa*) plus sequences downloaded from the GenBank database for the five species studied (each individual labeled with their GenBank accession number). Alignment length was 615 bp for 239 specimens. *Barbotinia oraniensis* was selected as the outgroup. Support for clades is indicated by the posterior probability values above branches.

Finally, the Bayesian tree built based on COI data only for 239 individuals from a number of countries shows approximately the same branch topographies, with *A. victrix* sister to *P. villosa* with 100% support, and both species sister to a clade formed by *A. brevis* and *A. castanea*. When more wasps from countries other than Belgium are included in the phylogeny, *A. brevis* is no longer monophyletic. Once again, *A. ramulifera* is the most distantly related species in this group (Figure 7).

The estimated evolutionary sequence divergence based on Kimura 2-parameter distances shows that *A. castanea* has the highest intraspecific variability in COI sequences whereas *A. ramulifera* shows the least intraspecific variation. When comparing between species, the most divergent species pair is *A. castanea* and *P. villosa*, with *A. castanea* and *A. brevis* constituting the most similar species pair (Table 1).

## 4. Discussion

Species of *Alloxysta* are very small and exhibit a smooth, shiny body surface. They are characterized by relatively few diagnostic characters, as is the case for many microhymenopterans. This lack of morphological features makes their identification very difficult, a fact that is compounded by the high number of species within the subfamily, which currently stands at 108 described species [33]. Many papers have been published with the aim of revising the type material of *Alloxysta* and other members of Charipinae [48,49,50,51,52,53,54].

A previous study showed that most species limits established within the Charipinae based on morphological features seemed correct [30]. This conclusion was supported by a combined analysis of molecular and morphological information. However, there are still complexes of species in this subfamily that deserve attention, mainly within *Alloxysta*, which is the most speciose and diverse charipine genus. Another study compared brachypterous with fully winged species of *Alloxysta* to determine whether sexual dimorphism of wing length and shape could be used as a character to distinguish species [31]. This analysis was based on morphological characteristics, but the need to test the usefulness of wing dimorphism to establish species limits using breeding and genetic analyses was also discussed. More recently, we employed genetic analyses to support the hypothesis that some brachypterous forms that were previously considered to be separate species are part of fully winged species [55].

One interesting result from this study is the fact that *Phaenoglyphis villosa* was found to be nested within *Alloxysta*, suggesting that it should be placed within the genus *Alloxysta*. This species was originally placed in the genus *Phaenoglyphis* based on morphological characters [32]. A previous molecular phylogeny of *Alloxysta* from Norway included *P. villosa*, but it was considered the outgroup [30]. Our study shows that, *P. villosa* is genetically more closely related to *A. victrix*. Therefore, the molecular data are in discordance with the morphological data. We recommend revising the morphological features used to characterize the genus *Phaenoglyphis* to make sure these features are robust enough and obtaining further molecular data for more *Phaenoglyphis* species, in particular the novel metazoan-level universal single-copy orthologs (metazoan USCOs) which hold much promise for species delimitation and taxonomy [56]. Specimens of *Phaenoglyphis* are usually captured in a much lower proportion than *Alloxysta* specimens in the field, which is the main reason why we know little about this genus. A possible reason for *P. villosa* being nested within *Alloxysta* is that some species within the genus *Phaenoglyphis* have fewer diagnostic features, such as notauli or scutellar foveae, including *P. villosa*, which does not have notauli present, and this is similar to *Alloxysta*. Additionally, *Phaenoglyphis villosa* has radial cells partially open, as well as two other *Phaenoglyphis* species (*P. china*: Ferrer-Suay and Pujade-Villar 2013, and *P. asiatica*: Ferrer-Suay and Pujade-Villar 2013). Finally, cryptic species and complexes of species are very common for Hymenoptera that are parasitic [57], making their study more complicated. The novel relationship uncovered here warrants further exploration in future studies. According to a previous study [58], there is a group of *Phaenoglyphis* species that lack these features, and they are close to other Charipinae species, suggesting an evolutionary tendency towards feature loss and a simpler structure.

The genetic distances between species of *Alloxysta* and *Phaenoglyphis* had not been determined previously. Some studies found that a genetic distance of 8.9% was enough to separate species of the genus *Diplolepis* (Cynipidae) [59] and *Ephedrus* (Braconidae) [60]. For the four *Alloxysta* and the one *Phaenoglyphis* species included in this study, interspecific genetic distances ranged from 12.4% to 16.4%, based on COI data. It is not surprising that *A. castanea* has the highest intraspecific variability given that this species is very abundant in multiple biogeographic regions [34]. Having high haplotype variability might have contributed to the successful colonization of new habitats. 

The three molecular markers included in our study, when combined, were useful in differentiating five Charipinae species. Each marker on its own was also able to separate the five species (not shown), although we are cautious not to draw conclusions from phylogenetic trees built based on as few base pairs as we retained after trimming the sequences for ITS2 and 16S. 

In conclusion, the phylogenetic relationships of *Alloxysta* and *Phaenoglyphis* are more complex than previously thought, and these genera likely need a deep revision. It is worth noting that when more *Alloxysta* and *Phaenoglyphis* species are included to build the subfamily phylogenetic tree, these other species might fit in between the five species considered in this study. Ideally, future work should increase the number of samples and the number of species, include genomic data (such as USCOs), and reconcile morphology and genetics to help establish the evolution of Charipinae species limits and relationships unambiguously. 

## Figures and Tables

**Table 1:** Mean pairwise distance matrix (Kimura 2-parameter distances ± standard deviation) for COI sequences within (on the diagonal) and between (below the diagonal) *Alloxysta* and *Phaenoglyphis* species.

	*A. castanea*	*A. victrix*	*A. ramulifera*	*A. brevis*	*P. villosa*
*A. castanea*	0.0247 ± 0.0329				
*A. victrix*	0.1452 ± 0.0017	0.0176 ± 0.0045			
*A. ramulifera*	0.1248 ± 0.0008	0.1409 ± 0.0009	0.0022 ± 0.0008		
*A. brevis*	0.1237 ± 0.0012	0.1367 ± 0.0011	0.1324 ± 0.0025	0.0035 ± 0.0035	
*P. villosa*	0.1640 ± 0.0012	0.1299 ± 0.0068	0.1431 ± 0.0093	0.1293 ± 0.0012	0.0145 ± 0.0113

## Data Availability

The original contributions presented in the study are included in the article and Supplementary Materials, further inquiries can be directed to the corresponding author.

## Supplementary Materials

The following supporting information can be downloaded at: https://www.mdpi.com/article/10.3390/insects15050354/s1, File S1: Fasta formatted alignment of 35 individual wasps in the Charipinae plus a cynipid gall wasp as outgroup, concatenating three genes, mitochondrial cytochrome oxidase I gene (COI), 16S ribosomal RNA encoding gene sequences (16S rDNA), and nuclear internal transcribed spacer 2 (ITS2 rDNA). Gaps in this alignment are not coded as separate partitions, the length of the alignment is 1374 base pairs; File S2: Fasta formatted alignment of 35 individual wasps in the Charipinae plus a cynipid gall wasp as outgroup, concatenating three genes, mitochondrial cytochrome oxidase I gene (COI), 16S ribosomal RNA encoding gene sequences (16S rDNA), and nuclear internal transcribed spacer 2 (ITS2 rDNA). Gaps are added to the data file as separate partitions, resulting in an alignment of 1471 base pairs; File S3: Fasta formatted alignment of 239 individual wasps in the Charipinae and a cynipid gall wasp as outgroup for mitochondrial cytochrome oxidase I gene (COI). Alignment length is 615 base pairs.
