# Supplementary material for: Molecular Data Confirm Interspecific Limits of Four Alloxysta and One Phaenoglyphis Species of Parasitic Wasps within the Subfamily Charipinae (Cynipoidea: Figitidae)
Source: Insects. 2024 May 14;15(5):354. doi: 10.3390/insects15050354 (PMC11121985; doi:10.3390/insects15050354)
Supplement: Supplementary file 1 [file insects-15-00354-s001.zip › insects-2974575-supplementary/Data-for-Mar/Supplementary Materials files.pdf]

Supplementary Materials for Interspecific limits within Charipinae (Cynipoidea: Figitidae), insights from molecular data

Mar Ferrer-Suay [1,*], Mariana Bulgarella [2], George E. Heimpel [3], Ehsan Rakhshani [4] and Jesús Selfa [1]

Supplemental Data File S1. Fasta formatted alignment of 35 individual wasps in the Charipinae plus a cynipid gall wasp as outgroup, concatenating three loci, mitochondrial cytochrome oxidase I gene (COI), 16S ribosomal RNA encoding gene sequences (16S rDNA), and nuclear internal transcribed spacer 2 (ITS2 rDNA). Gaps in this alignment are not coded as separate partitions, the length of the alignment is 1,377 base pairs.

Supplemental Data File S2. Fasta formatted alignment of 35 individual wasps in the Charipinae plus a cynipid gall wasp as outgroup, concatenating three loci, mitochondrial cytochrome oxidase I gene (COI), 16S ribosomal RNA encoding gene sequences (16S rDNA), and nuclear internal transcribed spacer 2 (ITS2 rDNA). Gaps are added to the data file as separate partitions, resulting in an alignment of 1,471 base pairs.

Supplemental Data File S3. Fasta formatted alignment of 239 individual wasps in the Charipinae and a cynipid gall wasp as outgroup for mitochondrial cytochrome oxidase I gene (COI). Alignment length is 615 base pairs.
